# Relationship between dental erosion and asthma medication in children: a systematic review

**DOI:** 10.21142/2523-2754-1301-2025-233

**Published:** 2025-03-03

**Authors:** André Alexis Díaz Quevedo, Diana Guadalupe Anaya Rubina, Carol Magaly Cárdenas Flores

**Affiliations:** 1 School of Dentistry, Universidad Cientifica del Sur. Lima, Peru. 100047903@cientifica.edu.pe 100053851@cientifica.edu.pe Universidad Científica del Sur School of Dentistry Universidad Cientifica del Sur Lima Peru 100047903@cientifica.edu.pe 100053851@cientifica.edu.pe; 2 Division of Pediatric Dentistry, School of Dentistry, Universidad Cientifica del Sur. Lima, Peru. ccardenasf@cientifica.edu.pe Universidad Científica del Sur Division of Pediatric Dentistry School of Dentistry Universidad Cientifica del Sur Lima Peru ccardenasf@cientifica.edu.pe

**Keywords:** Asthma, children, dental erosión, asma, erosión dental, niños

## Abstract

**Introduction:**

Dental erosion is an alteration that affects the integrity of teeth, which has several aetiologies. It is mentioned that asthma medications may be an etiologic factor; however, studies fail to clarify the possible association between these variables in children with asthma. Therefore, the purpose of this study will be to determine the relationship between dental erosion and asthma medication consumption in paediatric patients.

**Materials and methods:**

A systematic research was performed in databases such as MEDLINE (PubMed), Scopus, Web of Science, Scielo, Cochrane Library, Embase, LILACS and gray literature (Open Gray). Two researchers independently selected the articles according to the population, exposure, outcome, study design (PEOS) question using the Rayyan program. Newcastle-Ottawa scale was used to assess the risk of bias.

**Results:**

Six articles were included from 120 selected articles. The studies by Bairappan, Domenzain and Arafa studies show a higher prevalence of dental erosion in children with asthma medication as opposed to healthy children, whereas the studies by Dugmore and Rock, Alazmah and Rezende report a higher prevalence of erosive lesions in healthy children.

**Conclusions:**

Asthma medications are not a determining factor for the occurrence of erosive lesions in the teeth of paediatric patients with this systemic condition.

## RESUMEN

**Introducción:**

La erosión dental es una alteración que afecta la integridad de las piezas dentarias y tiene diversas etiologías. Se menciona que los medicamentos para el asma pueden ser un factor etiológico; sin embargo, los estudios no logran aclarar la posible asociación entre estas variables en niños con asma. Por ello, el objetivo de este estudio fue determinar la relación entre la erosión dental y el consumo de medicamentos para el asma en pacientes pediátricos.

**Materiales y métodos:**

Se realizó una búsqueda sistemática en las bases de datos MEDLINE (vía PubMed), Scopus, Web of Science, SciELO, Cochrane Library, Embase, LILACS y literatura gris (vía Open Gray). Dos investigadores independientes seleccionaron los artículos de acuerdo con la pregunta PEOS, en conjunto con el programa Rayyan. Se empleó la escala Newcastle-Ottawa para determinar el riesgo de sesgo.

**Resultados:**

De los 120 registros identificados, seis artículos fueron seleccionados con base en los criterios de inclusión. Los estudios de Bairappan, Domenzain y Arafa muestran la mayor prevalencia de la erosión dental en niños con medicación para asma, a diferencia de los niños sanos, mientras que los estudios de Dugmore y Rock, Alazmah y Rezende refieren una mayor prevalencia de lesiones erosivas en los niños sanos.

**Conclusión:**

El consumo de medicamentos para el asma no es un factor determinante para la aparición de lesiones erosivas en las piezas dentales en los pacientes pediátricos con esta condición sistémica.

## INTRODUCTION

Teeth are prone to structural changes due to the influence of the oral environment, in which a chemical wear process called dental erosion can develop with a group of specific factors. ^(^^1^^,^^2^ This tooth wear is caused by an interaction of the oral cavity with extrinsic or intrinsic acids. ^1^^-^^4^ The so-called acids can come from the diet, from the person's own gastric juices, or even certain medications used to treat diseases such as asthma. ^4^^-^^6^ Asthma is a chronic systemic condition characterized by airway obstruction due to an allergic or hypersensitivity reaction, which affects a considerable number of people worldwide. ^(^^7^^-^^9^ According to the International Study of Asthma and Allergies in Childhood, asthma is the most prevalent chronic condition in children overall. ^(^^10^ Although it is true that this pathology has no cure, however, asthma episodes can be controlled with inhalers such as bronchodilators (salbutamol) or steroids (beclomethasone). ^(^^11^^-^^13^ The frequency of use of these inhalers will depend greatly on exposure to the agents that cause asthmatic episodes and symptoms of the disease. ^7^^,^^11^

One study showed that the incidence of tooth erosion was higher in child patients suffering from asthma. ^14^ These results are consistent with other authors who evaluated the prevalence of this dental wear in children and adults with asthma problems. ^(^^15^^,^^16^ Furthermore, the asthma medications most associated with dental erosions were salbutamol, corticosteroids, or a combination of both in their inhaler presentation. ^6^^,^^16^^,^^17^ However, other studies did not find a significant relationship between the variables, mentioning that not all asthma medications are potentially erosive. ^(^^17^^-^^19^

There are studies in which they have investigated the relationship between dental erosion and the consumption of asthma medications in children, obtaining results that show that there is a relationship between these variables^20^^-^^24^, while others present the opposite. ^(^^17^^,^^18^^,^^25^ Therefore, the purpose of this study will be to determine the relationship between dental erosion and the consumption of asthma medications in paediatric patients, through a systematic review of published articles.

## MATERIALS AND METHODS

### Protocol and registration

This systematic review was registered in PROSPERO with the ID number CRD42021254243 and is under the guidelines of the Preferred Reporting Items for Systematic Review and Meta-Analysis (PRISMA). ^26^^)^

### Selection criteria

The review mainly included observational studies of case-control and cohort studies, published from 2000 to 2024. The study population should be in the age range of 3 to 17 years. Similarly, articles that mention the consumption of asthma drugs were included. 

Those articles that had as their study population patients with systemic disease other than asthma, patients with asthma and other associated comorbidity, studies that associate dental erosion with other factors such as eating disorders, or the consumption of acidic foods were excluded. Furthermore, studies with descriptive study design, studies with missing findings, and articles with a dubious methodological section were excluded. 

### Search strategies

A comprehensive bibliographic search strategy was carried out in the different databases such as MEDLINE (PubMed), Scopus, Web of Science, Scielo, Cochrane Library, Embase, LILACS and gray literature (Open Gray). 

The search strategy was limited to paediatric patients from January 2000 to May 2024, with no language restriction. In addition, several search terms related to the population, exposure, outcome, study design (PEOS) question were used in conjunction with MeSH terms and Boolean operators (AND, OR). Keywords such as "dental erosion", "tooth erosion", "asthma", "children" were used. The consolidated search was as follows: ("tooth erosion"[MeSH terms] OR "tooth erosion"[Title/Abstract] OR "dental erosion"[MeSH terms] OR "dental erosion"[Title/Abstract] OR "children"[MeSH terms] or "children"[Title/Abstract] or "asthma"[MeSH terms] or "asthma"[Title/Abstract]). Thus, a manual search was carried out systematically. This search strategy was adapted in the different databases. 

### Study selection

All selected articles were exported to the EndNote X9 program for the evaluation of each content of the published articles. Thereupon, the information was exported to the Rayyan program for the selection and exhaustive review of the articles found in the different databases. 

In the first stage, two reviewers (AADQ, DGAR) independently eliminated the duplicated articles using the Rayyan program, then carried out the selection of articles according to the title. Those articles that included the words that are related to the variables of the systematic review were selected.

In the second stage, both review authors independently analysed the abstracts of those selected articles to choose the appropriate studies for the third stage.

**Fig.1:**
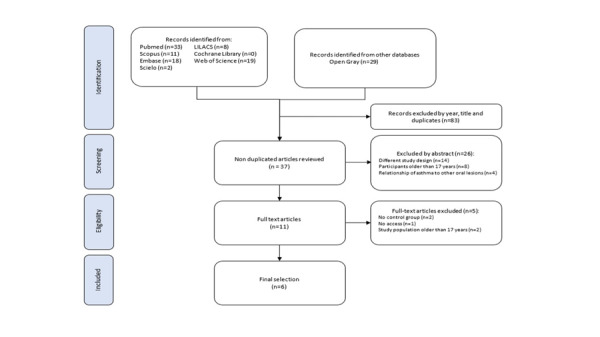
PRISMA ﬂow diagram detailing the study selection

In the third stage, both reviewers downloaded the articles in full text for examination and subsequent selection, considering the established inclusion and exclusion criteria (Fig. 1). During the process of selecting the articles, there was disagreement between the two reviewers, so the presence of a third reviewer was necessary. In addition, information was requested via email from an author to purchase the full-text article.

### Standardization process

With all the information from the Rayyan program, the criteria were standardized and the agreement observed among the evaluators was evaluated using the Kappa test. The concordance found between the two evaluators (AADQ, DGAR) was greater than 0,80. 

### Data extraction

Data extraction was performed by the 2 review authors (AADQ, DGAR). In this regard, with the help of the Microsoft Excel program, a table was created that allows the compilation of the data of the selected articles: Author/year of publication (1); title (2); objective (3); type of study (4); population (age and number of patients): deciduous dentition (5), mixed dentition (6), permanent dentition (7); Methodology: type of drug (8), time of consumption of the drug (9), consumption of sugars (10); Results: Dental erosion (11) and conclusions (12). The accuracy of data collection was confirmed by a third reviewer who was responsible for resolving discrepancies. 

### Data analysis and assessment

The information obtained was described in an orderly manner to develop evaluation tables and better observe the homogeneity of the information obtained from the selected articles. The data found were not homogeneous because the studies used different diagnostic methods for dental erosion, were done in different countries, and not all studies detail the measures of association and the types of asthma medications they used. Therefore, meta-analysis was not performed.

### Risk of bias

The risk of bias assessment of the selected observational studies was carried out by two review authors using the Newcastle-Ottawa scale (NOS) where each case-control and cohort study was assessed based on three domains: 1) Study group selection; 2) comparability of groups and 3) determination of exposure. ^(^^27^ According to the evaluation, each study will obtain a maximum score of 9 points. If the study score is seven to nine, the risk level will be low. A rating less than or equal to six will be high risk. ^27^

## RESULTS

### Study selection

A total of 120 articles were identified; after removing duplicates and some articles because they were outside the established search period and based on the title, 37 articles remained. The title and abstract of each article were independently assessed by the two reviewers. A further 26 articles were excluded because they did not meet the inclusion criteria. Five articles ^28^^-^^32^ were excluded due to missing findings, restricted access, and not considering a control group within the evaluation. A third author was requested to resolve discrepancies between the two reviewers regarding study selection. Finally, 6 articles met the inclusion criteria and were included for qualitative assessment (Figure 1).

### Included studies characteristics

Of the total number of articles found, 5 ^6^^,^^17^^-^^19^^,^^22^ were cohort studies and 120 was a case-control study. Regarding the sociodemographic context, Alazmah and Arafa *et al*. ^(^^18^^, 22)^ conducted the study in Saudi Arabia, while the studies by Bairappan *et al*., Dugmore and Rock, Rezende *et al*., and Domenzain *et al*. ^(^^6^^,^^17^^,^^19^^,^^20^ were conducted in India, England, Brazil, and Mexico, respectively. In relation to the age of the population, the studies by Dugmore and Rock, Alazmah, Rezende *et al*., Domenzain *et al*., and Arafa *et al*. *17-20, 22* evaluated similar populations in terms of age (3-12 years) and only the study by Bairappan *et al*. ^6^ evaluated adolescents aged 12 to 15 years. The study by Dugmore and Rock^17^ evaluated a more representative sample of 1753 participants, as opposed to the other studies^6^^,^^18^^-^^20^^,^^22^ which had 100, 180 or 228 participants. All studies^6^^,^^17^^-^^20^^,^^22^ have the characteristic of excluding patients with an associated comorbidity or if they had any other systemic condition other than asthma. The studies by Alazmah and Arafa *et al*. ^(^^18^^,^^22^ used the Smith and Knight Dental Wear Index as a diagnostic method, while Bairappan *et al*. ^(^^6^ used the dental erosion index from World Health Organization (WHO) oral health assessment form for children of the year 2013; Dugmore and Rock, and Rezende *et al*. ^(^^17^^,^^19^, the 1993 Children's Dental Health Survey Index in the United Kingdom; Domenzain *et al*. ^(^^20^, the Basic Erosive Wear Examination (BEWE) index. The studies by Bairappan *et al*., Dugmore and Rock, and Domenzain *et al*. ^(^^6^^,^^17^^,^^20^ were the only ones to evaluate the type of medication used by asthmatic patients, of which bronchodilators, corticosteroids and/or a combination of both stand out. The studies by Dugmore and Rock, and Domenzain *et al* ^*. (*^^17^^,^^20^ refer to the surfaces most affected by dental erosion, of which Dugmore and Rock ^17^ highlight the palatal surfaces of the upper teeth and the buccal surface of the lower teeth, while Domenzain *et al*. ^(^^20^ mention the palatal surfaces of the anterosuperior teeth and the occlusal surface of the lower teeth (Table 1).

**Table 1:** Data extraction table.

Author	Year	Country	Study design	Population age	Inclusion and exclusion criteria	Sample size	Diagnostic methods	Mention of medications	Most affected tooth surfaces
Abdulfatah Alazmah^18^	2021	Saudi Arabia	Cohort	3-12 years	Include children under the age of 12 with parental consent and without other systemic disease	100	Smith and Knight Tooth Wear Index	----	--------
Dugmore and Rock^17^	2003	England	Cohort	12 years	Include 12-year-olds from all state-maintained schools in Leicestershire and Rutland, selected based on the inclusion of one in five children in the respective school register	1753	Index from the 1993 UK Children´s Dental Health Survey	Corticosteroid Bronchodilators Cromolyn	Palatal surfaces of the upper teeth Buccal surface of the lower teeth
Domenzain *et al*. ^(^^20^	2021	Mexico	Cases controls	5-12 years	Exclude children with systemic disease other than asthma	228	BEWE Index (Basic Erosive Wear Examination)	-----	Palatal surfaces of the anterosuperior teeth Occlusal surface of the lower teeth
Rezende *et al*. ^(^^19^	2019	Brazil	Cohort	6-12 years	Include asthmatic children (registered in health units) and non-asthmatic children (registered with the Asthma program in health units)	228	Index from the 1993 UK Children´s Dental Health Survey	Bronchodilator Bronchodilator + Corticosteroid or Glucocorticoid + Systemic corticoids	-----
Bairappan *et al*. ^(^^6^	2020	India	Cohort	12-15 years	Asthmatic patients with another associated systemic disease and with medication other tan asthma are excluded	100	Tooth Wear Index (WHO 2013 oral health assessment form for children)	Bronchodilators Corticosteroids Bronchodilators + Corticosteroids Antihistamines + Bronchodilators Antihistamines + Bronchodilators + Corticosteroids	-----
Arafa *et al*. ^(^^22^	2017	Saudi Arabia	Cohort	4-12 years	Exclude children with serious diseases or other chronic systemic conditions other than asthma	180	Smith and Knight Tooth Wear Index	------	-----

UK, United Kingdom; WHO, World Health Organization

### Risk of bias assessment

Overall, of the total number of studies analysed, three were at high risk^(6^^,^^20^^,^^22^ and three were at low risk of bias. ^(^^17^^-^^19^ Bairappan *et al*., Dugmore and Rock, and Arafa *et al*. ^(^^6^^,^^17^^,^^22^ did not meet the criterion of representativeness of the exposed cohort. Only Dugmore and Rock´s study^17^^)^ met the criterion related to sufficiently long follow-up (2 years) for results. Regarding the study by Domenzain *et al*. ^(^^20^, it did not meet the adequate case definition and control selection (Table 2 and 3). 

**Table 2:** Risk of bias assessment for cohort studies.

Study		Selection			Comparability		Outcome		NOS Score
	Representativeness of the exposed cohort	Selection of the non-exposed cohort	Assessment of exposure	Demonstration that outcome of interest was not present at start of study	Comparability of cohorts on the basis of the design or analysis	Assessment of outcome	Was follow-up long enough for outcomes to occur	Adequacy of follow up of cohorts	
Dugmore and Rock^17^		*	*	*	*	*	*	*	7
Rezende *et al*. ^(^^19^	*	*	*	*	*	*		*	7
Arafa *et al*. ^(^^22^		*	*	*	*	*		*	6
Abdulfatah Alazmah^18^	*	*	*	*	*	*		*	7
Bairappan *et al*. ^(^^6^		*	*	*	*	*		*	6

NOS, Newcastle-Ottawa Scale

**Table 3:** Risk of bias assessment for case control studies.

Study	Selection				Comparability	Outcome			NOS Score
	¿Is the case definition adequate?	Representativeness of the cases	Selection of controls	Definition of controls	Comparability of cases and control son the basis of the design or analysis	Assessment of exposure	Same method of ascertainment of exposure	Non-response rate		
Domenzain *et al*. ^(^^20^		*		*	*	*	*	*	6

NOS, Newcastle-Ottawa Scale

### Relationship between medication exposure and presence of dental erosion

Of the total number of articles selected, the higher prevalence of dental erosion in asthmatic children compared to children without asthma was highlighted in the studies by Bairappan *et al*., Alazmah, Domenzain et al. and Arafa *et al*. ^(^^6^^,^^18^^,^^20^^,^^22^ with a total of 44%, (p<0.05), 24% (p>0.05), 36.8% (p<0.05, OR=2.018 (1.05 - 3.85)) and 40% (p<0.05) respectively. However, the study by Alazmah^18^ concluded that there is no correlation between asthma and the presence of dental erosion, unlike the studies by Bairappan *et al*., Domenzain *et al.* and Arafa *et al*. ^(^^6^^,^^20^^,^^22^ which concluded that there was a significant association between asthma and the presence of dental erosion. On the other hand, the studies by Dugmore and Rock, and Rezende *et al*. ^(^^17^^,^^19^ showed the highest prevalence of dental erosion in children without asthma, with an estimated 58.9% (p>0.05, OR=1.05) and 32.14% (p>0.05) respectively (Table 4). 

**Table 4:** Association between medication exposure and dental erosion or outcome.

Author	Sample size	Nº of children with asthma	Nº of children without asthma	Outcome		Dental erosion prevalence percentage (%)		p value	Measure of association	General conclusion
				CA	CWA	CA	CWA			
Abdulfatah Alazmah^18^	100	50	50	12	9	24	18	> 0.05	-	There is no correlation between asthma and the presence of tooth erosion.
Dugmore and Rock^17^	1753	268	1331	158	794	58.9	59.7	0.89	OR=1.05	There is no association between asthma and the presence of dental erosion. Medications are not potentially erosive
Domenzain *et al*. ^(^^20^	228	76	152	28	34	36.8	22.4	0.02	OR=2.018 (1.05-3.85)	Significant association between asthma and the presence of dental erosion
Rezende *et al*. ^(199^	228	112	116	36	44	32.14	37.9	0.36	-	There is no association between the presence or absence of tooth erosion and the types of asthma medication
Bairappan *et al*. ^(^^6^	100	50	50	22	9	44	18	0.043	-	Significant association between asthma and the presence of dental erosion
Arafa *et al*. ^(^^22^	180	120	60	48	8	40	13.3	≤0.001	-	Significant association between asthma and the presence of dental erosion

CA, children with asthma; CWA, children without asthma; OR, odds ratio

## DISCUSSION

The present systematic review evaluated the association between asthma medication and the presence of dental erosion in asthmatic children aged 3 to 17 years in articles published in the databases. Three studies supported the association of drug use and the presence of tooth erosion in children with asthma. ^(^^6^^,^^20^^,^^22^ Of the three articles in favor, two studies follow a cohort design^6^^,^^22^ and one case-control design. ^20^ The overall sample size of the studies evaluated is very heterogeneous. In the study by Dugmore and Rock^17^ the sample size was 1753; while in the studies by Bairappan *et al*. ^(^^6^ and Alazmah ^(^^18^ the sample size was 100 children. The observed differences in sample size mean that the association between the variables is not consistent. Likewise, the distribution of children with asthma and healthy children was not homogeneous, unlike the studies by Bairappan *et al*. ^(^^6^ and Alazmah^18^ who considered an equitable distribution of the population. Despite having an equitable distribution, the study by Bairappan *et al*. ^(^^6^ found a significant association between drug consumption and the presence of erosive lesions, while Alazmah's study^18^ found no association between the variables. 

One of the reasons for the inconsistency of the association between the variables studied is that most of the studies^6^^,^^17^^,^^18^^,^^20^ used different diagnostic methods for dental erosion except for the studies by Arafa *et al*. ^(^^18^ and Alazmah ^22^ who used the same diagnostic index of Smith and Knight. In addition, the studies by Dugmore and Rock, and Rezende *et al*. ^(^^17^^,^^19^ agreed on the use of the index from the 1993 Children's Dental Health Survey in the United Kingdom. The difference is that the Smith and Knight index provides a score from 0 to 4 on the 4 surfaces of all teeth, while the BEWE index rates each tooth with a score from 0 to 3 based on the highest score of each sextant and then adds them together to obtain a final score that indicates the severity of tooth erosion. ^(^^33^^,^^34^ The index of the 1993 Children's Dental Health Survey in the United Kingdom is similar to the dental erosion index from WHO oral health assessment form for children of the year 2013, because they assess tooth erosion according to the depth of the lesion, whether it encompasses enamel, dentin or pulp. ^(^^6^^,^^17^^)^ However, they differ in that the first index from 1993 compiles the area of the tooth affected by erosion. ^(^^17^ In this sense, these tools are a source of heterogeneity between studies. Although the studies by Alazmah and Arafa *et al*. ^(^^18^^,^^22^ were conducted in the same country, they differ in relation to the association of the variables studied. The study by Arafa *et al*. ^(^^22^ found a significant association between asthma medication consumption and the development of dental erosion, while Alazmah's study^18^ found no association. Another difference found in the analysis is that some of the studies^18^^,^^20^^,^^22^ do not specify the type of medication used in the patients, as well as the time of application. However, they report that asthma medication has a reducing effect on pH and salivary flow, which suggests that it is a factor for the development of alterations in tooth structure such as dental erosion. ^(^^6^^,^^17^^,^^22^^,^^35^^,^^36^ In relation to the dental surfaces most affected by dental erosion, the literature shows a higher incidence at the level of the palatal surfaces of the upper incisors and the occlusal surfaces of the lower molars. ^(^^1^^,^^37^ In the review, only the studies by Dugmore and Rock, and Domenzain *et al*. ^(^^17^^,^^20^ observed that the palatal and occlusal surfaces were the most affected.

The erosive effect of asthma control medications has been evaluated by several studies. ^(^^5^^,^^38^ The pH of these drugs is less than 5,5, and they chemically dissolve tooth enamel if daily administration is frequent. ^(^^39^ If the medication is administered on a long-term basis, it is associated with a greater possibility of developing alterations at the level of the dental structure, such as dental erosion. ^(^^38^ However, in the available evidence, not all studies had much information about the medications, such as the time of exposure and the doses administered. Therefore, it is recommended in future research to detail more information about asthma medication to determine its influence.

Dental erosion is influenced by many factors, and asthma medication can contribute to its onset, especially in children who are the most susceptible population to asthma.^40^^)^ It is therefore important to carry out adequate follow-up in these patients, both in general and oral health. The initial anamnesis will help us determine if the presence of dental erosion is caused by factors such as the consumption of asthma medications, diet, the patient's hygiene or another associated factor.^41^ It is also suggested to rinse with water after taking asthma medication to control pH levels.^39^

### Limitations

Due to the observational nature of the studies, there is a high risk of bias, which means that the results should be interpreted cautiously, as they do not allow for conclusions about causality. Studies that found a significant association between the variables are particularly vulnerable to bias. In contrast, authors who oppose the association tend to have a lower risk of bias, suggesting that this discrepancy could indicate a reason to question the association between the variables in light of potential biases. Moreover, the studies assessed confounding factors such as brushing frequency ^18^^,^^19^, salivary characteristics ^6^^,^^22^, and parental education level. ^(^^18^^,^^19^ Only two studies did not specify whether confounding factors were considered. ^(^^17^^,^^20^ These factors can significantly influence the outcomes, aligning with a systematic review that indicates the intervention group cannot be directly compared to the control group at baseline. ^(^^42^ Additionally, the studies could not be included in a meta-analysis due to heterogeneity from various issues, such as the lack of standardization in the diagnostic criteria for dental erosion, the studies conducted in different countries, the absence of association measures, and insufficient information regarding asthma medications. This variability suggests that the findings concerning the possible relationship between dental erosion and asthma medication are compromised, representing a limitation in drawing any accurate conclusions.

## CONCLUSION

The consumption of asthma medications is not a determining factor for the occurrence of dental erosive lesions in paediatric patients with this systemic condition. Both variables do not present a significant association due to the heterogeneity of the studies. Although the consumption of these medications influences the appearance of erosive lesions, there are other factors that play an important role in this oral condition. Therefore, future research is required to provide more information to clarify the relationship asthma medication has with tooth erosion.
